# Dietary Polyphenol and Methylsulfonylmethane Supplementation Improves Immune, DAMP Signaling, and Inflammatory Responses During Recovery From All-Out Running Efforts

**DOI:** 10.3389/fphys.2021.712731

**Published:** 2021-08-31

**Authors:** Brian K. McFarlin, David W. Hill, Jakob L. Vingren, John H. Curtis, Elizabeth A. Tanner

**Affiliations:** ^1^Applied Physiology Laboratory, Department of Kinesiology, Health Promotion, and Recreation, College of Education, University of North Texas, Denton, TX, United States; ^2^Department of Biological Sciences, College of Science, University of North Texas, Denton, TX, United States

**Keywords:** optimized curcumin, pomegrante extract, MSM, PAXgene, Nanostring, inflammation

## Abstract

Nutritional ingredients with defined mechanisms of action can be useful in the recovery of the body from the physical demands of a habitual training plan. The purpose of this study was to determine the effect of dietary supplementation with optimized curcumin, pomegranate ellagitannins, and MSM (R + MSM) on immune-associated mRNA during early recovery (i.e., up to 8 h post-exercise) following all-out running efforts (5-km, 10-km, and 21.1-km). Subjects (*N* = 14) were randomized to either a supplement (R + MSM) or a control group using an open label design. The study was completed over a period of 31-day prior to a scheduled half-marathon race. Venous blood samples were collected into PAXgene tubes at baseline, subsequent samples were collected at 2, 4, and 8 h after each running effort. A 574-plex mRNA Immunology Array (NanoString) was measured for each sample and ROSALIND^®^ Advanced Analysis Software was used to examined changes in 31 annotated immune response pathways and specific mRNA changes. The greatest change in immune pathways occurred at 2 h (GSS > 3) followed by 4 h (GSS 2–3) and 8 h (GSS 1–2). R + MSM was associated with an increase in innate immunity (CAMP, LTF, TIRAP, CR1, IL1R1, CXCR1, PDCDILG2, and GNLY) and a blunted/smaller increase in damage-associated molecular pattern (DAMP) signaling/inflammation (TLR4, TLR5, S100A8, S100A9, and IFP35). We also found changes in immune-associated mRNA that have not been previously linked to exercise recovery (SOCS1, SOCS2, MME, CECAM6, MX1, IL-1R2, KLRD1, KLRK1, and LAMP3). Collectively these results demonstrate that supplementation with a combination of optimized curcumin, pomegranate ellagitannins, and methylsulfonylmethane resulted in changes that may improve biological recovery from all-out running efforts.

## Introduction

Participating in aerobic exercise training programs results in beneficial adaptations in cardiovascular, metabolic, muscular, and immune function (Ruegsegger and Booth, 2018). Worldwide, many people use running as the exercise modality of choice (Lee et al., 2014). While exercise training is generally beneficial, recovery after a bout of exercise varies as a function of running speed and distance. Our laboratory and others have examined the efficacy of dietary polyphenols (e.g., curcumin, ellagitannins, punicalagins) and organosulfur compounds (e.g., methylsulfonylmethane) to improve recovery from exercise sessions (Kawanishi et al., 2013; Ammar et al., 2016; McFarlin et al., 2016; Tennent et al., 2017; Tanner et al., 2020a,b). To our knowledge, the published literature has not established the effectiveness of any particular combination of polyphenols and organosulfur compounds that is effective across a range of exercise doses. This knowledge would be particularly useful when incorporating these supplements into a long-term training plan as such plans generally include varying duration and intensities of exercise.

Mechanistically, dietary polyphenols and organosulfur compounds are considered good candidates as components to support exercise-training because their endogenous activity is very similar to that of non-steroid anti-inflammatory drugs, and they have no known side effects (Davis et al., 2007; Kawanishi et al., 2013; Ammar et al., 2016; Butawan et al., 2017; Danesi and Ferguson, 2017; Tennent et al., 2017; Gupte et al., 2019). Some of the known actions include an upregulation of antioxidant defense (e.g., superoxide dismutase, catalase, and glutathione peroxidase) and a blunting of cyclooxygenase-2 (COX-2) and nuclear factor kappa beta (NF-κb) activity (Davis et al., 2007; Kawanishi et al., 2013; Ammar et al., 2016; Butawan et al., 2017; Danesi and Ferguson, 2017; Tennent et al., 2017; Gupte et al., 2019). These actions broadly impact immune function and inflammation, resulting in more effective exercise recovery and reduced risk of infection during long-term training. The present study sought to expand the knowledge base regarding supplementation with these compounds, which have the potential to improve biological exercise recovery by altering the immune response.

Buttner et al. (2007) completed one of the first studies to examine the effect of acute exercise on white blood cell gene expression. In this study, our laboratory sought to expand on this initial work by examining post-exercise white blood cell mRNA expression under conditions under conditions and with a regime of dietary supplementation. Specifically, the present study, which sought to expand existing knowledge by combining high-throughput mRNA testing (574-plex immune-associated mRNA array) with the latest ROSALIND^®^ bioinformatics techniques to measure change in 31 specific immune-response pathways. We designed the present study to target mRNA changes, which permits a greater number of identifiable outcomes than simply examining protein change. Identified pathways were used to as a guide to identify significant changes in mRNA that have the potential to impact immune function, inflammation, and thus biological exercise recovery. The purpose of this study was to determine the effect of dietary supplementation with curcumin, pomegranate ellagitannins, and MSM on immune-associated mRNA during early recovery (up to 8 h post-exercise) following all-out running efforts over 5-km, 10-km, and 21.1-km.

## Materials and Methods

### Participants

The current study protocol was approved by the Institutional Review Board at the University of North Texas and all aspects of the study were conducted in accordance with the Declaration of Helsinki. All participants gave verbal and written informed consent prior to beginning the supplementation routine or performing any exercise testing and data collection. Potential participants were excluded if they reported diagnosed chronic disease, regular supplementation with curcumin/turmeric and/or pomegranate and/or MSM, habitual intake of pain relievers (i.e., ibuprofen, acetaminophen, aspirin), and/or contraindications to exercise. This study used a novel approach, with NanoString ROSALIND^®^ technologies, to investigate over 500 outcomes that define immunological, DAMP signaling, and inflammatory responses after three different exercise challenges. Because of the uniqueness of the study, both in terms of the outcomes and the exercise challenges, data were not available to perform an *a priori* power analysis to determine an acceptable sample size. Rather, we used a convenience sample of individuals who were already in training for a half-marathon race. Sample size was limited by the need to perform exercise tests on all participants in a very narrow time frame in terms of time of day (between 0600 and 0900) and date (∼3 weeks before the half-marathon and ∼2 weeks before the half-marathon). Fourteen individuals (Table 1; Subject Characteristics) were randomized to either a supplement group (*n* = 6) or a control group (*n* = 8). Two additional participants dropped out of the study due to a scheduling conflict. Individuals who participated in this study were already committed to running a half-marathon within the next 30 days and had already been training prior to joining the study. The average 30-day training data for the two groups are found in Table 1. The sample size for the present study was determined based on previous studies from our laboratory using a similar supplement and exercise model (Gary et al., 2019; Tanner et al., 2020a,b). These previous studies included between 10 and 20 subjects, which was similar to the enrollment of the present study. Subject characteristics of the two groups were statistically compared using a *t*-test and the *p* < 0.05 level of significance.

**TABLE 1:** Participant characteristics, training, and performance data.

	R + MSM (*N* = 6)	Control (*N* = 8)

Participant characteristics		
Gender (# F)	3	3
Age (y)	39 ± 6	40 ± 7
Height (cm)	176 ± 8	177 ± 7
Weight (kg)	78.9 ± 12.1	76.5 ± 11.1
BMI	25 ± 3	24 ± 3
% Fat	27 ± 8	24 ± 9
VO_2max_ (ml/kg/min)	43.9 ± 8.9	46.8 ± 9.6
Anaerobic threshold (% of max)	68% ± 13%	71% ± 11%
HR Max (bpm)	185 ± 14	184 ± 12
**30-d training data**		
Total distance (miles)	49.5 ± 5.2	51.3 ± 5.0
Total duration (h)	19.2 ± 1.1	20.5 ± 0.9
Total caloric expenditure (kcal)	6,899 ± 756	7,198 ± 708
**Running performance data**		
5-km Time Trial (min)	25.6 ± 0.9	26.0 ± 1.0
5-km HR Max (bpm)	187 ± 4	186 ± 5
10-km Time Trial (min)	54.3 ± 0.9	54.9 ± 1.9
10-km HR Max (bpm)	186 ± 5	188 ± 3
21.1-km Run (min)	135.6 ± 4.9	141.6 ± 10.7
21.1-km HR Max (bpm)	166 ± 7	172 ± 6

*Participants supplemented with R + MSM = Restoridyn^®^ (Optimized Curcumin and pomegranate ellagitannins; Verdure Sciences; Indianapolis, IN, United States) and methylsulfonylmethane (OptiMSM^®^; Bergstrom Nutrition; Vancouver, WA, United States). Open label control participants did not consume a supplement. PAXgene blood samples were obtained at 2 h, 4 h, and 8 h after the all-out running efforts of 5-km, 10-km, and 21.1-km. The 5-km (Day 10) and 10-km (Day 17) were completed during the lead-up to anoutdoor half marathon race (Day 31). The 5-km and 10-km time trials were completed indoors on a motorized treadmill at a similar time (0600 to 0900) as the half marathon race. Values represent mean ± SEM. There was no significant difference between groups.*

### Supplementation Schedule

The project team was blinded to supplementation assignment throughout the study, including during all running trials, sample analysis, and statistical analyses, to ensure an unbiased approach to data collection and interpretation of the findings. Given the “open label” nature of this study, subjects did not consume a traditional placebo when they were assigned to the control group. The following supplement schedule was specific to the treatment group (R + MSM). During days 0–27, the daily dose of the experimental supplement was 1,000 mg of a proprietary synergistic polyphenol blend (optimized curcumin and pomegranate ellagitannins; Restoridyn^®^; Verdure Sciences Corp; Indianapolis, IN, United States) and 500 mg of methylsulfonylmethane (OptiMSM^®^; Bergstrom Nutrition; Vancouver, WA, United States). During days 0–27, participants also consumed an additional daily dose (booster dose) within 1 h of completing a run of 9.6 km or longer (5.3 ± 3.3 total booster doses consumed per subject). The 5-km and 10-km laboratory time trials were completed at 3 and 2 weeks, respectively, prior to the 21.1-km road race. During days 28, 29, and 30, the dose was doubled (Restoridyn^®^ 2000 mg/d; OptiMSM^®^ 1000 mg/d), participants were instructed to taper their running distance and to discontinue use of booster doses. The 21.1-km road race was completed on Day 31. The two components of the experimental supplement have anti-inflammatory/antioxidant actions and they have documented safety records, making them good candidates for the use in an exercise recovery model (Patel et al., 2008; Butawan et al., 2017; Gupte et al., 2019). Dosing was consistent with previously published studies from our laboratory and designed to meet the demands of a 21.1-km running race (Tanner et al., 2020a,b).

Supplement doses were provided 16-day at a time using a daily blister pack format. When the participant returned to the laboratory, they were asked to return their previous blister pack to track supplement compliance. Participants were asked to notify a member of the study staff about any missed doses. We noted that for this particular study, our subject compliance for the planned doses was >95%.

### Self-Prescribed Exercise Training Program

The present study was designed to follow participants who had already been training to run a half-marathon race, which they intended to run within the next 30 days. As such, subjects were asked to maintain their individual pre-determined training regimen and to log all running sessions using an “app” (MapMyRun; Under Armour; Baltimore, MD, United States). Using this approach, data regarding the session time, distance run (GPS), and intensity (heart rate via wrist telemetry) were collected (Table 1; 30-d training data). There was no significant difference between the quantity of training for participants in the R + MSM or control groups.

### Running Efforts

As a lead-up to the half-marathon, subjects were asked to complete two all-out running efforts in the laboratory, 3 weeks (5-km) and 2 weeks (10-km) prior to the half-marathon. A motorized treadmill (4Front; Woodway; Waukesha, WI, United States) was used for testing in the laboratory. The half-marathon was completed at a local, outdoor race event. All three running trials were completed between 0600 and 0900 to avoid circadian effects on performance (Hill, 2014) and immune response (McFarlin et al., 2003). Performance data included time-to-completion and maximal heart rate (Table 1; Running performance data). The present study was not focused on comparing the RNA response to the different running efforts and thus extensive performance data were not collected. Also, a thorough comparison of the RNA response to the efforts would have required a much larger sample size to delimit order effects and individual performance differences, which was not the focus of the present study. The sample size of the present study was small for performance measurement comparisons and thus we found no significant difference between R + MSM and control (Table 1; Running performance data).

### Blood Collection, RNA Isolation, and Analysis

One venous blood sample was obtained during the initial visit to the laboratory, after participants had completed all paperwork and provided consent, and before the supplementation regimen began. On the days when participants performed a running test (i.e., 5-km, 10-km, or 21.1-km), a venous blood sample was obtained prior to (Pre) exercise, and 2, 4, and 8 h post run. An automated complete blood count (CDS Medonic; Plantation, FL, United States) was used to monitor hydration status (via hemoglobin and hematocrit), which was similar at all time points. Whole blood was collected from an antecubital vein in the arm into a 10 mL (2.5 mL blood + 6.5 mL of PAXgene solution) RNA stabilizing vacutainer (PreAnalytiX, Hombrechtikon, Switzerland). Vacutainers were mixed by inversion and stored at −20°C for 24 h, prior to being stored at −80°C until RNA isolation and analysis. Total RNA was extracted from PAXgene blood using a commercially available RNA isolation kit (PAXgene Blood miRNA kit; PreAnalytiX) and an automated system (QIAcube; Qiagen, Hilden, Germany). Isolated RNA was concentrated to 35 ng/μL using a spin column kit prior to NanoString analysis. Concentrated RNA was analyzed using a 574-plex Human Immunology Panel v 2.0 (nCounter; NanoString, Seattle, WA, United States) and raw image counts (RCC files) were obtained using a Sprint Profiler (NanoString) following a 16-h hybridization assay. In addition to the targets, specific internal positive/negative controls and housekeeping mRNA (EEF1G, RPL19, PPIA, HPRT1, TBP, TUBB, POLR1B, ABCF1, SDHA, GUSB, G6PD, ALAS1, POLR2A, GAPDH, and OAZ1) were included with every RNA sample. An additional volume of blood was collected to measure liver enzymes and basic metabolic parameters using an automated chemistry analyzer (data not shown, no significant difference between groups or time points). The analysis reliability measures included image quality (99 ± 5%), binding density (1.09 ± 0.47), and positive control linearity (0.98 ± 0.01). These reliability measures were within the ranges established by the manufacture.

### ROSALIND^®^ NanoString Gene Expression Methods

Data were analyzed by ROSALIND^®^^1^ with a Hyperscale architecture developed by ROSALIND, Inc. (San Diego, CA, United States). The ROSALIND^TM^ platform aims to bring better collaboration and data science to genomic data interpretation so that organizations and institutes can harness the potential of high-value data from DNA sequencing to microarrays and mass spectrometry outputs, while reducing costs and increasing productivity. Read Distribution percentages, violin plots, identity heat maps, and sample multi-dimensional plots were generated as part of the quality control step. Normalization, fold changes, and *p*-values were calculated using criteria provided by NanoString. ROSALIND^®^ follows the nCounter^®^ Advanced Analysis protocol of dividing counts within a lane by the geometric mean of the normalizer probes from the same lane. Housekeeping probes to be used for normalization were selected based on the geNorm algorithm as implemented in the NormqPCR R library (Perkins et al., 2012). Fold changes and significance score (*p*-value) were calculated using the fast method as described in the nCounter^®^ Advanced Analysis 2.0 User Manual (NanoString). Significant *p*-values (*p* < 0.05) were adjusted for multiple genetic comparisons using the Benjamini–Hochberg method of estimating false discovery rates (Ferreira, 2007). Gene clustering (based on direction and type of all signals on a pathway, the position, role and type of every gene) of differentially expressed genes determined using the Partitioning Around Medoids method and the fpc R library (Hennig, 2019).

### ROSALIND^®^ Pathway and Meta-Analysis

Hypergeometric distribution was used to analyze the change of immune response pathways. The topGO R library (Alexa, 2019) was used to determine local similarities and dependencies between GO terms in order to perform Elim pruning correction. Several database sources were referenced for immune response pathway analysis, including InterPro (Mitchell et al., 2019), NCBI (Geer et al., 2010), MSigDB (Subramanian et al., 2005; Liberzon et al., 2011, 2015), REACTOME, and WikiPathways (Slenter et al., 2018). Directed global significance scores (GSS) were calculated relative to a set of background genes (baseline samples) relevant for the experiment. Within this analysis both significance and directional response (i.e., upregulated or downregulated) of GSS for a given immune response pathway was determined. A total of 31 immune response pathways were examined to determine the effectiveness of supplement (R + MSM) as a function of exercise recovery time (2, 4, and 8 h) according to combined exercise and isolated exercise effort (i.e., 5-km, 10-km, and 21.1-km). Radar plots were generated to compare immune response pathways as a function of directed GSS. Immune pathways of interest were used as a guide for ROSALIND^®^ Meta-Analysis to identify clusters of specific mRNA changes that occurred with R + MSM compared to control. Within this approach we excluded clusters that only had an exercise effect but were not differentially expressed between R + MSM and control as this was not the purpose of the present study. mRNA were expressed as log2 fold change from baseline in order to normalize the response to a 0 center and indicate direction of expression (i.e., upregulated vs. downregulated).

## Results

### Effect of R + MSM on Exercise Recovery: Pathway Approach

**FIGURE 1:**
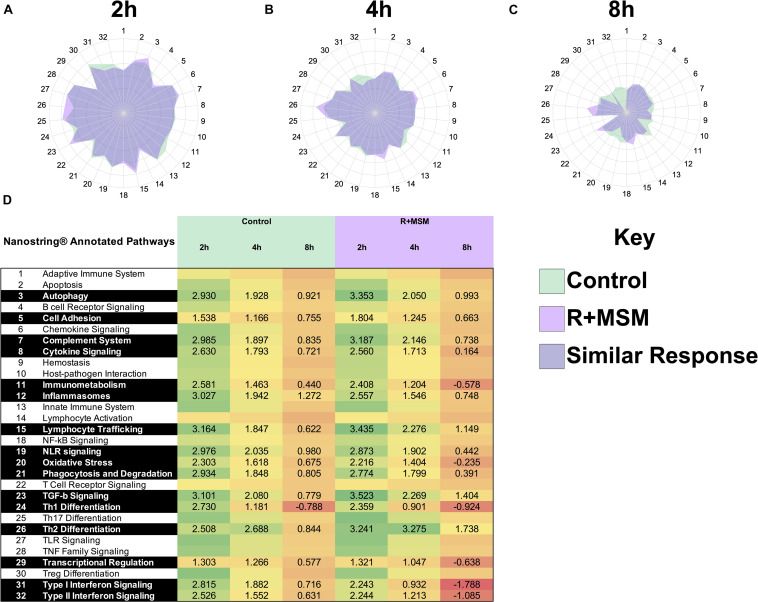
Immune response pathway comparison between combined supplementation with a proprietary synergistic polyphenol blend (optimized curcumin and pomegranate ellagitannins; Restoridyn^®^; Verdure Sciences Corp.; Indianapolis, IN, United States) and methylsulfonylmethane (OptiMSM ^®^; Bergstrom Nutrition; Vancouver, WA, United States) and Control (no supplement) following recovery from all-out running efforts (5-km, 10-km, and 21.1-km). PAXgene blood samples were collected at 2 h **(A)**, 4 h **(B)**, and 8 h **(C)** after the all-out running efforts over a period of 31-day. Total RNA was isolated and analyzed using a 574-plex mRNA Immunology array (NanoString nCounter). ROSALIND^®^ Advanced Analysis Software was used to conduct a pathway analysis using NanoString Annotations to calculate directed global significance scores (GSS) for 31 immune response pathways. Supplement (R + MSM) is represented by light purple, control is represented by green, and dark purple represents areas where R + MSM and control had similar responses. The greatest magnitude of GSS response was at 2 h and the lowest was at 8 h. Significantly impacted immune response pathways and associated GSS are presented in **(D)**.

The complex nature of this data set required a stepwise approach to interpret the various findings. Thus, initially, we collapsed data across the three exercise tests and examined the effect of R + MSM vs. control at the post-exercise time points (2, 4, and 8 h). Differential pathway analysis was conducted using the baseline sample (obtained prior to any supplementation) as a comparison for all subsequent samples. Pre-exercise measurements were excluded from this analysis because the control (no supplement) sample did not have any significantly changes from baseline, thus preventing a pathway comparison at that time point. Directed GSS for 31 immune response pathways was used to generate radar plots to compare control and R + MSM at 2 h (Figure 1A), 4 h (Figure 1B), and 8 h (Figure 1C). We also included a heat map with significant GSS scores (Figure 1D) as an alternate way to report the data associated with pathway changes. The greatest magnitude of immune response pathway change occurred at 2 h (GSS > 3.0) followed by 4 h (GSS 2–3) and 8 h (GSS 1–2). Significance pathways that were either upregulated or downregulated with treatment compared to control were further identified (Table 2). Nine immune response pathways were changed with R + MSM compared to control at 2 h. Examining the pattern of change in these pathways at 4 and 8 h revealed the following patterns: two pathways remained upregulated (Lymphocyte Trafficking and Th2 Differentiation), three pathways reduced to the same level as control (Autophagy, Cell Adhesion, and Th1 Differentiation), and four were downregulated (Complement, Inflammasome, Type 1 IFN, and Type 2 IFN). We also identified six pathways that were downregulated with R + MSM at 4 or 8 h that were not changed at 2 h (Oxidative Stress, Immunometabolism, Cytokine Signaling, NLR Signaling, Phagocytosis, and Transcriptional Regulation). Collectively, across all three exercise recovery time points, we found that 16 of 31 immune response pathways examined were upregulated or downregulated with R + MSM compared to control, all reflecting a “better” recovery response in the R + MSM group. These specific pathways cluster around two main immune outcomes that are important to exercise recovery: innate immune response and DAMP signaling/inflammation.

**TABLE 2:** Response of GSS pathways in R + MSM compared to Control.

Immune response pathway	2 h	4 h	8 h
Autophagy	↑	↔	↔
Cell adhesion	↓	↔	↔
Complement	↑	↑	↓
Inflammasome	↓	↓	↓
Lymphocyte trafficking	↑	↑	↑
Th1 differentiation	↓	↔	↔
Th2 differentiation	↑	↑	↑
Type 1 IFN-gamma	↓	↓	↓↓
Type 2 IFN-gamma	↓	↓	↓↓
Oxidative stress	↔	↓	↓↓
Immunometabolism	↔	↓	↓↓
Cytokine signaling	↔	↔	↓
NLR signaling	↔	↔	↓
Phagocytosis	↔	↔	↓
Transcriptional regulation	↔	↔	↓↓
TGF-beta	↔	↔	↑

*Participants supplemented with R + MSM = Restoridyn^®^ (Optimized Curcumin and pomegranate ellagitannins; Verdure Sciences; Indianapolis, IN, United States) and methylsulfonylmethane (OptiMSM^®^; Bergstrom Nutrition; Vancouver, WA, United States). Open label control participants did not consume a supplement. PAXgene blood samples were obtained at 2 h, 4 h, and 8 h after the all-out running efforts of 5-km, 10-km, and 21.1-km. PAXgene whole blood was used to isolate total RNA that was then analyzed using a 574-plex mRNA Immunology Array (Nanostring nCounter). Directed global significance scores (GSS) were calculated by a ROSALIND^®^ Advanced Analysis pathway enrichment technique. Arrows indicate the effect of R + MSM compared to Control: the same in R + MSM and Control (↔); higher or much higher in R + MSM (↑ or ↑↑); and lower or much lower in R + MSM (↓ or ↓↓).*

### Effect of R + MSM on Exercise Recovery After Different Running Efforts: Pathway Approach

**FIGURE 2:**
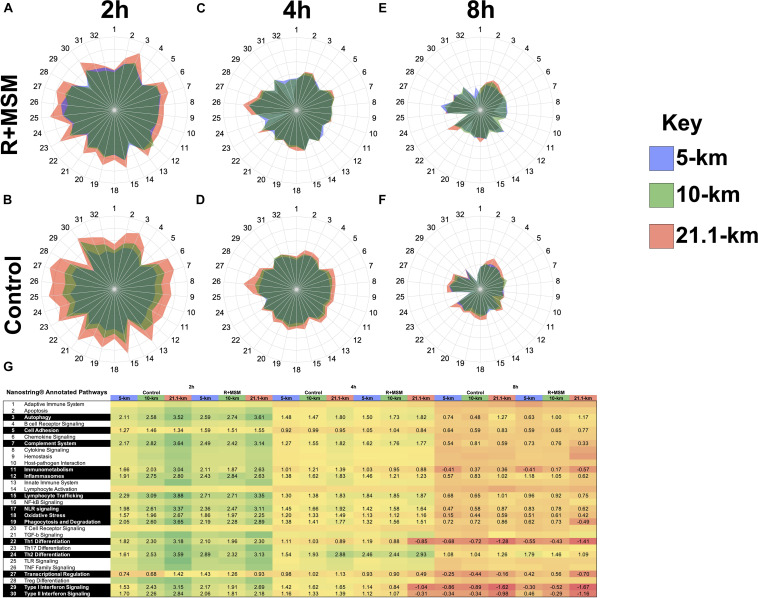
Immune response pathway comparison as a function of supplement type, running effort length, and post-exercise time. Combined supplementation with a proprietary synergistic polyphenol blend (optimized curcumin and pomegranate ellagitannins; Restoridyn^®^; Verdure Sciences Corp.; Indianapolis, IN, United States) and methylsulfonylmethane (OptiMSM^®^; Bergstrom Nutrition; Vancouver, WA, United States) (R + MSM) and Control (no supplement) following recovery from all-out running efforts (5-km, 10-km, and 21.1-km). PAXgene blood samples were obtained at 2 h, 4 h, and 8 h after the all-out running efforts of 5-km, 10-km, and 21.1-km. Total RNA was isolated and analyzed using a 574-plex mRNA Immunology array (NanoString nCounter). ROSALIND^®^ Advanced Analysis Software was used to conduct a pathway analysis using NanoString Annotations to calculate directed global significance scores (GSS) for 31 immune response pathways as a function of supplement (R + MSM vs. Control) and exercise recovery time (2, 4, and 8 h) as follows: R + MSM 2 h **(A)**, R + MSM 4 h **(C)**, R + MSM 8 h **(E)**, Control 2 h **(B)**, Control 4 h **(D)**, Control 8 h **(F)**. 5-km is represented by a light blue shading, 10-km is represented by light green shading, and 21.1-km is presented by light orange shading. The greatest magnitude of GSS response was at 2 h and the lowest was at 8 h. Significantly impacted immune response pathways and associated GSS are presented in **(G)** as a function of supplement type, post-exercise time, and running effort length.

Our initial analysis was aimed at identifying an immune profile response for R + MSM compared to control during exercise recovery regardless of exercise effort. Using the global immune response pathways as a road map, we focused on these 16 pathways when comparing both supplement and exercise efforts as factors in the model. Control and R + MSM were compared at 2 h (Figures 2A,B), 4 h (Figures 2C,D), and 8 h (Figures 2E,F). The greatest magnitude of immune response pathway change occurred at 2 h (>3.0 GSS) followed by 4 h (2–3 GSS) and 8 h (1–2 GSS). Further that those time points the was a progressive change in pathway response as a function of effort length (i.e., 5-km vs. 10-km vs. 21.1-km). Changes within running effort length was also realized as a function of supplement (R + MSM vs. Control). With R + MSM, 5-km (Figure 2A) and 10-km (Figure 2C) were similar and less pronounced than the GSS response for 21.1-km (Figure 2E). Control had a different response where 5-km (Figure 2B) had the smallest magnitude of GSS response followed by 10-km (Figure 2D) and 21.1-km (Figure 2F). At 4 h, R + MSM resulted in nearly identical GSS responses (Figure 2C), while 21.1-km was still elevated above 10-km and 5-km with control (Figure 2D). By 8 h the remaining GSS pathway changes were small and similar between R + MSM (Figure 2E) and control (Figure 2F). We also included a heat map with significant GSS scores (Figure 2G) to report the data associated with pathway changes in an alternate format. Further interpretation of these results revealed two details that guided further analysis and interpretation of these results: (1) running effort influences immune response pathways and (2) R + MSM appears to be effective regardless of the running effort. Given these findings we focused our meta-analysis on identifying mRNA that clustered to change as a function of R + MSM vs. control.

### Effect of R + MSM on Exercise Recovery: Meta-Analysis

**FIGURE 3:**
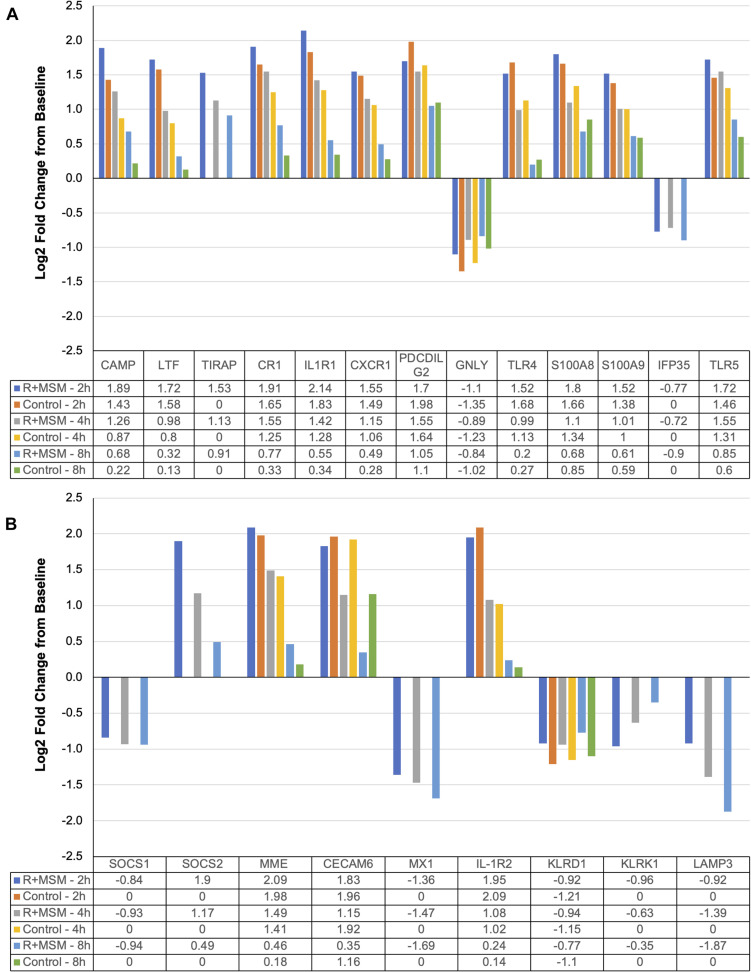
Specific immune response mRNA (Log2 fold change from baseline) changes compared as a function of combined supplementation with a proprietary synergistic polyphenol blend (optimized curcumin and pomegranate ellagitannins; Restoridyn^®^; Verdure Sciences Corp.; Indianapolis, IN, United States) and methylsulfonylmethane (OptiMSM^®^; Bergstrom Nutrition; Vancouver, WA, United States) and Control (no supplement) following recovery from all-out running efforts (5-km, 10-km, and 21.1-km). PAXgene blood samples were obtained at 2 h, 4 h, and 8 h after the all-out running efforts of 5-km, 10-km, and 21.1-km. Total RNA was isolated and analyzed using a 574-plex mRNA Immunology array (NanoString nCounter). ROSALIND^®^ Advanced Analysis Software was used to calculate Log2 fold change using the baseline samples from each condition. ROSALIND^®^ Meta-Analysis was used to identify mRNA that were significantly changed between R + MSM and Control. **(A)** Includes mRNA that have been previously reported to change following acute bouts of exercise (similar to the present study). **(B)** Includes mRNA that are not known to be affected by acute exercise, but may have a role in post-exercise immunity.

The two pathway analyses yielded a set of 96 specific mRNA (17% of all mRNA targets examined) that were significantly upregulated or downregulated with R + MSM compared to control. ROSALIND^®^ meta-analysis was conducted to compare clustered mRNA that were changed with R + MSM compared to control. Initial data screening excluded 74 mRNA that were changed in a similar manner between R + MSM and control (exercise effect). Since the purpose of this study was not to evaluate the effects of exercise alone, we excluded these mRNA from further discussion (data not shown). We identified 22 candidate mRNA (Figure 3) that were altered with R + MSM compared to control following exercise. After searching the available literature, we determined that 13 of our target candidates (Figure 3A) had previously been reported to be affected by exercise, whereas nine target candidates (Figure 3B) with known biological actions have not been previously reported to be altered by exercise. The 13 mRNA candidates (Figure 3A) were clustered relative to: innate immune response (CAMP, LTF, TIRAP, CR1, IL1R1, CXCR1, PDCDILG2, and GNLY) and DAMP signaling/inflammation (TLR4, TLR5, S100A8, S100A9, and IFP35). The remaining nine mRNA (Figure 3B) that have not been linked to acute exercise response were associated with regulation of cytokine signaling, DAMP signaling, and apoptosis (SOCS1, SOCS2, MME, CECAM6, MX1, IL-1R2, KLRD1, KLRK1, and LAMP3). We did identify that some these mRNA may respond to chronic training in clinical disease population; however, those populations and exercise model do not match well to the design of the present study, making further interpretation difficult.

## Discussion

By combining high-throughput genetic techniques with ROSALIND^®^ bioinformatics, we were able to identify 16 immune response pathways and 22 subsequent mRNA candidates whose exercise recovery response was significantly altered by supplementation with optimized curcumin, pomegranate ellagitannins, and MSM compared to control. Further regardless of exercise effort, R + MSM altered both immune response pathways and specific mRNA compared to control. Using a meta-analysis approach, we were able to determine that the mRNA associated with the 16 immune response pathways altered with R + MSM tended to cluster around two main biological outcomes that are important for exercise recovery: innate immune function and DAMP signaling/inflammation. Both of these pathways have implications not only for post-exercise infection prevention, but also for biological recovery of exercise-induced muscle damage. Per the key purpose of the study, the remainder of the discussion is dedicated to discussing the changes that resulted in a differential manner with R + MSM compared to control at a given time and differentially normalized to baseline measurements.

We found that supplementation with optimized curcumin, pomegranate ellagitannins, and MSM prevented suppression of mRNA associated with the innate immune system that was observed following exercise in the control subjects. Supplementation was associated with an overall significant increase in the expression of two antimicrobial peptides: cathelicidin antimicrobial peptide (CAMP; 60% upregulation) and lactotransferrin (LTF; 63% upregulation), which protect of the skin and lungs from bacteria (Murakami et al., 2002; Tjabringa et al., 2005; Hao et al., 2019). Murakami et al. (2002) previously reported an increase of CAMP release from sweat immediately after exercise. In the present study, while CAMP and LTF expression were elevated at 2 h than 4 or 8 h, their expression was consistently higher with R + MSM. With respect to the innate immune response, we observed that R + MSM created differential expression compared to control for chemokine CXC motif receptor 1 (CXCR1; 8 and 43% less downregulation at 4 and 8 h), complement component 3b/4b receptor 1 (CR1; 31% upregulation across 2, 4, and 8 h), granulysin (GNLY; 12% less downregulation across 2, 4, and 8 h), and programmed cell death 1 ligand 2 (PDCDILG2; 9% less upregulation across 2, 4, and 8 h). We also found an increased expression of toll-interleukin 1 receptor domain containing adaptor protein (TIRAP) for R + MSM following exercise but no change was found for control. There are published reports of exercise effects on CR1 (Pascual and Schifferli, 1992; Thomsen et al., 1992), GNLY (Li et al., 2007), and PDCDILG2 (Schenk et al., 2021); however, we report novel effects of R + MSM supplementation and exercise on CXCR1 and TIRAP. The supplement-induced blunting of the decline in CXCR1 was associated with an increased capacity to deploy neutrophil extracellular traps (NET), which are critical to neutrophil-mediated innate immune response to influenza pneumonia (Rudd et al., 2019) and bacteria (Sahley et al., 2007). CR1 expression impacts the ability of erythrocytes to move immune complexes to the macrophage system for elimination (Pascual and Schifferli, 1992). Only a single published study has examined erythrocyte CR1 in the context of exercise (Thomsen et al., 1992) and it reported that erythrocyte CR1 expression was not changed immediately or 2 h after strenuous exercise (60 min of cycling at 75% of VO_2__*max*_). Our findings differed in that we observed that 31% exercise-induced decline in CR1 expression (i.e., in controls) was blunted with R + MSM. While the effect of exercise on TIRAP is not fully known, it is known that TIRAP is a critical component of monocyte response to gram-negative bacteria and thus increased expression post-exercise with supplementation may translate into an improved innate immune response to bacterial challenge (Ciesielska et al., 2021). Granulysin (GNLY) is one of three granzymes found in NK cells and it has been previously reported to be elevated in healthy, physically active individuals (Li et al., 2007). This combined with the present finding indicates that supplementation may improve post-exercise NK cell innate immune response. Schenk et al. (2021) reported that endurance exercise (50-min of moderate intensity exercise) was associated with a reduction in T-cell Programmed cell death 1 ligand 2 (PD1) expression 1 h after exercise. The present study extends these findings by reporting that PD1 expression continued to be decrease at 2, 4, and 8 h into recovery and that R + MSM supplementation reduced the overall decline by 9% compared to control. Our laboratory and others have speculated that strenuous exercise increases post-exercise T-cell apoptosis, and it is reasonable to speculate that this increase may be explained by reduced PD1 expression (Navalta et al., 2010a,b). We also found reductions in SOCS1 and LAMP3 with treatment, but not control. To our knowledge, no published studies have reported acute effects of exercise on either SOCS1 or LAMP3, so the fact that we observed a reduction with R + MSM is a novel and unknown if it is a potentially beneficial finding. Together, the above-mentioned observed R + MSM alterations in mRNA demonstrate significant post-exercise improvements in innate immunity, including antimicrobial defense, immune complex trafficking, neutrophil function, monocyte function, NK cell function, and T-cell resistance to apoptosis. These findings may translate into not only a reduction in post-exercise infection risk with R + MSM, but also increased capacity to biologically respond to post-exercise sterile inflammation (McFarlin et al., 2016; Tanner et al., 2020a,b), which has implications for muscle recovery.

Our laboratory has spent considerable effort examining the relationship between exercise recovery and inflammation (McFarlin et al., 2006, 2007, 2016; Luk et al., 2016; Tanner et al., 2020a,b). The findings of the present study further the existing knowledge regarding post-exercise DAMP signaling/inflammation. Specifically, we observed that R + MSM compared to control was associated with increase in interleukin 1 receptor type 1 (IL1R1), a decrease in toll-like receptor 4 (TLR4), an increase in toll-like receptor 5 (TLR5), a decrease in S100 calcium binding protein A8 (S100A8), and a decrease in S100 calcium binding protein A9 (S100A9) compared to control at various time points after exercise. We also found a decrease in interferon-induced protein 35 (IFP35) with R + MSM, but no change was found for control. Changes in IL1R1, TLR4, and TLR5 are interrelated due to their capacity to respond to DAMPs and production of pro-inflammatory cytokines and mediators. Although IL1R1, TLR4, and TLR5 share a similar signaling pathway, their respective stimuli differ. An elevated IL1R1 would be indicative of an improved response to IL-1 allowing for better innate immune response, whereas TLRs are pattern recognition receptors sensitive to DAMPs. When R + MSM resulted in a 40% increase in IL1R1, a 13% decrease in TLR4, and a 22% increase in TLR5 greater than control. A supplement-induced reduction in TLR4 is consistent with anti-inflammatory actions in physically active individuals previously reported by our laboratory (Flynn et al., 2003; McFarlin et al., 2004a, 2006). TLR5 is negatively correlated to low-grade inflammation in the gut of mice (Chassaing et al., 2014), suggesting that it might track inflammation in an opposite manner than TLR4. In the present study, supplementation with optimized curcumin, pomegranate ellagitannins, and MSM reduced TLR4 and increased TLR5, which may translate to an overall decrease in propensity to post-exercise inflammation.

Observed changes in S100A8, S100A9, and IFP35 expression were particularly noteworthy because each is a DAMP commonly released in response to sterile inflammation or muscle injury (Willoughby et al., 2003; Mooren et al., 2006; Xiahou et al., 2017; Tanner et al., 2020a) and all signal through TLR4 (Ibrahim et al., 2013). R + MSM resulted in a 63 and 60% reduction in S100A8 and S100A9, respectively, between 2 and 8 h compared to a 49 and 57% reductions in controls. To our knowledge, only a single published study has examined the circulating concentration of S100A8 and S100A9 in response to exercise; that study reported that S100A8 and S100A9 remain elevated for 24 h after running a marathon (Mooren et al., 2006). The present exercise effect may have differed given that S100A8 and S100A9 declined between 2 and 8 h. The response of another DAMP, IFP35, was also blunted in the R + MSM group, which is noteworthy because it is known to signal through TLR4 (Xiahou et al., 2017). Collectively, findings of reductions in TLR4, SA100A8, SA100A9, and IFP35, and an increase in TLR5 support the notion that combined optimized curcumin, pomegranate ellagitannins, and MSM supplementation was associated with reduced post-exercise inflammation. Based on previous research, it is known that reduced post-exercise inflammation (like that observed with use of the R + MSM supplement in the present study) translates into a better functioning recovery process and a more rapid return to activity.

With the advent of new biomarker measurement techniques such as the ones used in the present study, our understanding of the exercise-recovery response and how to improve it is still evolving. A key finding of the present study was that using a supplement that combined optimized curcumin, pomegranate ellagitannins, and MSM resulted in post-exercise recovery responses that should translate into an improvement in readiness to return to subsequent exercise training sessions. While the present study includes novel findings regarding the potential of R + MSM supplementation to improve post-exercise immune function and recovery, it is not without limitations. The sample size in this study was not considered to be large. Nevertheless, we were able to clearly differentiate between responses of the experimental and control groups. Of course, with a larger sample size, we may have been able to identify more differences between response of the two groups. Therefore, in future work from our laboratory, we will attempt to replicate and expand on the current findings. Another potential limitation of the present study was related to dosing; however, the dosing was designed to match the anticipated physiological stress of run training (Tanner et al., 2020a,b). Despite the design limitation, the present study identified RNA who may be linked to the *in vivo* actions of R + MSM post-exercise. One limitation the present that we did not fully realize until after the study was complete is that the number of days that the supplement had been consumed may confound the ability to determine the effect of R + MSM on exercise dose (i.e., 5-km vs. 10-km vs. 21.1-km). It is our goal to address this limitation in the design and completion of a future experiment. As such, our dosing was designed to replicate how these supplements might be used by recreational exerciser in real life settings. While the pathways studied in the present study are not considered part of the somatic nervous system, it is plausible that there may have been an unrealized placebo effect in the R + MSM group. Future work might seek to further examine the effect of different durations and intensities of exercise on the mRNA responses within the same subjects.

Our laboratory has explored exercise recovery and the role of the immune system for nearly two decades (McFarlin et al., 2003, 2004b, 2005, 2009, 2013, 2016, 2017; Tanner et al., 2020a). In this work, we have attempted to understand the immune response and evaluate potential treatment options. The key findings of the present study support the notion that supplementation with optimized curcumin, pomegranate ellagitannins, and MSM has great potential as a recovery tool due to its potential biological impact. We observed no negative effects associated with supplementation but a capability to improve the innate immune response and reduce inflammation early in the exercise recovery process (2–8 h post exercise). These effects were noted across different race distances, at three different time points in the last month of preparation for a half-marathon race, meaning that these supplements might be useful for habitual consumption as part of a comprehensive exercise training plan. Research is needed to further refine dosing patterns with respect to a specific exercise stimulus.

## Data Availability Statement

The original contributions presented in the study are included in the article/supplementary material, further inquiries can be directed to the corresponding author.

## Ethics Statement

The studies involving human participants were reviewed and approved by the University of North Texas Institutional Review Board. The patients/participants provided their written informed consent to participate in this study.

## Author Contributions

BM contributed to the study design, interpretation, and manuscript preparation. JV and DH contributed to the interpretation and manuscript preparation. JC and ET contributed to the sample collection and analysis. All authors contributed to the article and approved the submitted version.

## Conflict of Interest

The authors declare that the research was conducted in the absence of any commercial or financial relationships that could be construed as a potential conflict of interest.

## Publisher’s Note

All claims expressed in this article are solely those of the authors and do not necessarily represent those of their affiliated organizations, or those of the publisher, the editors and the reviewers. Any product that may be evaluated in this article, or claim that may be made by its manufacturer, is not guaranteed or endorsed by the publisher.

## Footnotes

1
https://rosalind.onramp.bio/
